# Diabetes Mellitus as a Risk Factor in Glaucoma’s Physiopathology and Surgical Survival Time: A Literature Review

**DOI:** 10.5005/jp-journals-10008-1190

**Published:** 2016-02-02

**Authors:** Lívio Costa, João Paulo Cunha, Duarte Amado, Luís Abegão Pinto, Joana Ferreira

**Affiliations:** Consultant, Department of Ophthalmology, Centro Hospitalar de Lisboa Central, Lisbon, Portugal; Consultant, Department of Ophthalmology, Centro Hospitalar de Lisboa Central; Faculty of Medical Sciences, New University, Lisbon Portugal; Consultant, Department of Ophthalmology, Centro Hospitalar de Lisboa Central, Lisbon, Portugal; Professor, Department of Ophthalmology, Centro Hospitalar de Lisboa; Institute of Pharmacology and Neurosciences, Faculty of Medicine, Lisbon University, Lisbon, Portugal; Consultant, Department of Ophthalmology, Centro Hospitalar de Lisboa Central; Faculty of Medical Sciences, New University, Lisbon Portugal

**Keywords:** Diabetes mellitus, Fibroblasts and cytokines, Glaucoma, Glaucoma surgery, Healing process, Risk factors.

## Abstract

Glaucoma is a multifactorial condition under serious influence of many risk factors. The role of diabetes mellitus (DM) in glaucoma etiology or progression remains inconclusive. Although, the diabetic patients have different healing mechanism comparing to the general population and it has a possible-negative role on surgical outcomes.

This review article attempts to analyze the association of both diseases, glaucoma and DM, before and after the surgery.

The epidemiological studies, based mainly in population prevalence analyzes, have shown opposite outcomes in time and even in the most recent articles also the association remains inconclusive. On the contrary, the experimental models based on animal induced chronic hyperglycemia have shown an important association of both diseases, explained by common neurodegenerative mechanisms.

Diabetic patients have a different wound healing process in the eye viz-a-viz other organs. The healing process is more and it results in lower surgical survival time, higher intraocular pressure (IOP) levels and, therefore, these patients usually need more medication to lower the IOP. Both randomized and nonrandomized retrospective and experimental molecular studies have shown the association between DM and glaucoma.

Further studies are needed to get better explanations about outcomes on more recent surgical procedures and with the exponential use of antifibrotics.

**How to cite this article:** Costa L, Cunha JP, Amado D, Pinto LA, Ferreira J. Diabetes Mellitus as a Risk Factor in Glaucoma’s Physiopathology and Surgical Survival Time: A Literature Review. J Curr Glaucoma Pract 2015;9(3):81-85.

## INTRODUCTION

Glaucoma is a multifactorial condition. This article reviews the possible association between diabetes mellitus (DM) and intraocular pressure (IOP)/primary open-angle glaucoma (POAG). With this purpose, the authors have analyzed the laboratorial and experimental studies, the first and the most recent epidemiological articles and some retrospective randomized and non-randomized studies that have shown the outcomes in newly evolved glaucoma surgeries.

Multiple risk factors for the disease etiology and progression were extensively reviewed for this review article. We could find a statically significant association to higher age, black race, IOP > 21 mm Hg or with fluctuations, positive family history of glaucoma in a first degree relative and central corneal thickness (CCT). Each of these factors has a clear association with glaucoma etiology, even its progression.^1-3^

Diabetes mellitus is not a well understood risk factor and its association to IOP or POAG is still controversial. It would be good to re-evaluate the present status of this association for better understanding of the etiology and/ or the progression.^1-4^

## EXPERIMENTAL STUDIES

The individual POAG and DM physiological mechanisms are well known.

The glaucoma etiology is based on higher IOP levels resulting in biomechanical alterations on the lamina cribrosa structure, more in the location of higher fragility (inferior and superior neuroretinal rim). The ischemia is also an important contributor in the process, apparently because of the altered blood flow and autoregulatory vascular mechanisms.^1-3,7,10^

The individual changes in chronic hyperglycemia are essentially vascular, under the systemic endothelial cell dysfunction, oxidative stress and lipid glycation.^4,5^

The epidemiological studies remain inconclusive, but the experimental articles attempt to explain the biochemical mechanisms that link both the diseases.^5-10^

The laboratory-induced chronic hyperglycemia studies have shown a neurodegenerative mechanism affecting both neural and glial cells, impaired axonal transport and collagenous tissue remodeling on the trabecular meshwork and lamina cribrosa, similar to POAG.^5,6,8-10^

In glaucoma, there is impaired retrograde axonal transport with decreased production of neurotrophic factors, such as brain-derived growth neurotrophic factor (BDNF). Similarly, animal eyes with experimental-induced hyperglycemia have lower levels of these neurotrophic factors which are necessary for the ganglion cells apoptosis. The latter laboratorial evidence, suggest that neural and glial degeneration occur before the vascular alterations.^11-13^

When we look at the effect of IOP on the trabecular meshwork and lamina cribrosa, the remodeling of colla-genous and extracellular matrix by the transforming growth factor-B (TGF-B) and connective tissue growth factor, it turns out that these structures are more susceptible to additional stress. Again, in the experimental DM models, researchers were able to induce the growth of the same factors, which resulted in the structural alteration of the connective tissue.^11-13^

The latest analyzes on neurodegenerative mechanisms have shown an additional ganglion cell loss caused by DM in glaucoma patients. The already vulnerable ganglion cells in glaucoma eyes are under an additional stress in hyperglycemic conditions.^11-13^

## EPIDEMIOLOGICAL STUDIES

The experimental animal studies have shown molecular and cellular common mechanisms relating DM and IOP/ glaucoma, but even the recent epidemiological articles remain inconclusive.^14,15^

Reviewing the first experiences on this field, the evidence has suggested higher IOP and glaucoma incidence in diabetic cohort patients.^16-19^ The first evidence in Amstrong and Becker’s prevalence studies showed two to three times higher incidence of elevated IOP and POAG incidence in diabetes patients. Becker et al have shown additional results of increased tensional values secondary to corticoids, larger cup/disk ratio or higher susceptibility to field loss. In opposition, Bankes et al have not gotten these conclusions in their prevalence trials.^16-19^

The large population studies, such as Beaver Dam study, Rotterdam study or Blue Mountains Eye study have shown an association between DM and the higher IOP or development of POAG.^20,21,26,27^

The Rotterdam Study, for example, has shown that patients with DM had an overall rise in mean IOP of 0.31 mm Hg and an increased presence of POAG or even, the presence of serum glucose levels > 10 mmol/l was associated with a higher mean IOP and higher ratio to glaucoma of 2.82.^20,21^

Other studies (Framingham Eye study or Baltimore Eye Survey) do not corroborate this hypothesis.^22,23,25^

Differently, the European Glaucoma Prevention Study (EGPS) and the Ocular Hypertension Treatment Study (OHTS) have found that diabetes protects the patients with high IOP levels against the progression to glaucoma. But the authors have stated that the results were under multiple bias and the protective effect was denied.^24^

But searching the most recent results, the question and the answers remain unanswered. Coleman et al^28^ suggest that diabetes may be associated with open-angle glaucoma progression.^28^ Ellis et al failed to confirm the association.^15^ Leske et al analyzed the predictors for long-term progression in glaucoma and demonstrated the role of vascular factors, but they did not consider diabetes in particular.

The most controversial results came with the new Rotterdam study. The disparity in the studies could be due to lack of the same standardized methodologies or the statistics approaches.^20,21^

The clinical, structural and functional criteria to diagnose glaucoma have changed with time and the use of new guidelines have led to a reduced number of patients in the most recent analyzes. Even, the outcome parameters to diagnose or measure diabetes are different. The earlier studies have even used self-report to classify the diabetic or the control groups, certainly that has created bias.^20,21^

With the change in guidelines and criteria to diagnose glaucoma, the group in the recent Rotterdam study has reduced in size, and thus it turned the statistical significance absolutely inferior.^20,21^

While the clinical investigations based on the epide-miological and prevalence have been trying to prove or disprove this association, the new scientific approaches are trying to link the use of antidiabetic medication to reduce the risk for glaucoma.^29^

An experimental study presented in Annual Meeting of the Association for Research in Vision and Ophthalmology (ARVO) 2014 has shown metformin is able to reduce the risk of development of POAG. This relation was found to be dose-dependent and the reduction was 0.01% higher for each 1 gm more in metformin dose (p < 0.001). The patients with diabetes with cumulative doses more than 1110 gm at 2 years had a 25% lower risk of developing POAG, compared to a nondiabetic. Further studies are needed to understand the mechanism how the antidiabetic medications modulate the effect of hyperglycemia on diabetic patients and how this protects to glaucoma development.^29^

## IS DIABETES A RISK FACTOR TO GLAUCOMA SURGICAL OUTCOMES?

Literature search suggests that diabetics have usually higher IOP values in the immediate postoperative period and also on the longer follow-up time. Another important conclusion is the worst surgical survival time.^30-33^

The surgical outcomes have been extensively studied for classic trabeculectomy and argon laser trabeculoplasty (ALT), but the literature is sparse when we look for newer procedures, such as the nonpenetrating sclerectomy or drainage devices.^30-33^

In Advanced Glaucoma Intervention Study (AGIS), the authors have shown a three times higher (2.83, 1.88-4.36, p < 0.001) prevalence of trabeculectomy or ALT failure than in that patients without diabetes.^31^

The earlier retrospective and randomized studies have analyzed the group of patients with diabetes without stratifying them as per disease duration or stage. Literature has a huge diversity of articles considering these conclusions in varied group of patients, such as proliferative diabetic retinopathy, no proliferative diabetic retinopathy in progression or diabetes without retinopathy.^30-33^

Recently, Law et al^30^ used a cohort of diabetic patients without retinopathy to compare the outcomes of classic trabeculectomy with adjuvant Mitomycin C (MMC) after 6 months and over a longer period (1-8 years) of follow-up.^30^ This study confirmed lower IOP in both short and long follow-up periods. The survival time of surgery in control group was higher, but the difference between groups was not significant.^30^

Mariotti et al^34^ have reported in ‘long-term outcomes and risk factors for failure with the Express glaucoma drainage device ’ a higher failure in group of patients with diabetes, non-caucasian race or previous glaucoma surgery. The complete success rate decreased from 83% at 1 year to 57% at 5 years follow-up in control group. Differently, in group with diabetes, the result at 1 year was 63% and impaired to 42% at 5 years.^34^

Stephen et al examined the repair outcomes in drainage devices after the first exposure and they have listed the factors liable to the surgery failure: black race, DM, increase number of glaucoma medications before shunt implantation, a history of multiple glaucoma laser procedures and combination of an initial aqueous shunt implantation with another surgery.^35^

Diabetic patients were three times more likely to undergo a repeat intervention than the no diabetic patients and the period between the first and second intervention was lesser.^36^

Since the main analyzes of surgical outcomes have shown worse results in diabetic group, for all the surgeries including the classic trabeculectomy with or without antifibrotic agents or nonpenetrating surgeries, laser procedures or shunts implantation, we have to understand why this is happening? Probably its the difference in postoperative wound-healing process. Another possible mechanism is the vasculature abnormality associated with ischemia.^30,34-36^

## WOUND-HEALING PROCESS IN CHRONIC HYPERGLICEMIA

Diabetes is responsible for systemic changes on the vascular endothelial cells in all organs, more in micro-vascular structures, such as heart, kidney and eye. The general scar formation and wound healing in these patients are the big task in diabetes evolution. Under high glycemic serum levels the fibroblastic response in the wound is usually impaired and the total healing happens latter than in nondiabetic patients.^37-41^

The clinical practice has shown that opposite happens in the eye. The scar formation in subconjunctival and Tenon’s capsule, after the glaucoma surgery, is more in diabetics. The prognosis is worse because of the impaired survival time of trabeculectomy bleb.^37-41^

Browning et al have suggested that human Tenon’s capsule fibroblasts (hTCF) from eye have the same action as fibroblasts from the nonocular tissues. Analyzes of cytokine concentration in aqueous humor and vitreous revealed an increased concentration of transforming growth factors [transforming growth factor-p2 (TGF-P2) and platelet-derived growth factor (PDGF)-BB]. The higher proliferation on eye scar tissue is secondary to the activation of fibroblasts because of elevated cytokines.^40^

Kottler et al^38^ found evidence of significant contribution of TGF-beta 2 and an additional effect of TGF-beta in the scar formation after glaucoma procedures. So, TGF-beta could be another key to the adjunctive treatment.

Denk et al^39^ have shown a strong evidence that TGF-beta-isoforms induce the transformation of fibroblasts on myofibroblasts and the PDGF-isoforms are essential in Tenon’s capsule growth.

Another experimental article suggests role of pathways involving new TGF-beta factors and specific inhibitor signals. This shows the huge potential of combination therapies.

## CONCLUSION

Diabetic patients with POAG have an additional mechanism of damage on lamina cribrosa and trabecular mesh-work and they have relatively higher IOP.

Experimental laboratory results using animal-induced chronic hyperglycemia have shown that glaucoma patients with diabetes are more vulnerable to optic disk damage. But, the prevalence studies in last 40 years remain inconclusive to clarify the association.

Another important fact is the increased risk of failure of the glaucoma surgery in diabetics. These patients have higher IOP levels after surgery and need more antiglaucoma medications. In diabetic patients, more scar tissue forms in the subconjunctival tissue and this closes the surgical fistula. Higher concentrations of cytokine in anterior segment of diabetic patients accounts for marked cicatrization by inducing an activation of fibroblasts.
